# Study on the morphological characteristics and rotational alignment axis of placement plane of the tibial component in total knee arthroplasty for hemophilia-related knee arthritis

**DOI:** 10.1186/s13018-022-03176-4

**Published:** 2022-06-14

**Authors:** Ru Feng, Qigang Zhong, Liujie Zheng, Houlong Ye, Dasheng Luo, Mingyang Ding, Nanyu Pang, Jiale Li, Yunfeng Yao

**Affiliations:** Department of Orthopedic Surgery, Second Affiliated Hospital of Anhui Medical University, Heifei, 230601 China

**Keywords:** Total knee arthroplasty, Morphological variation, Rotational alignment axis, Hemophilia arthritis, Reference landmark

## Abstract

**Background:**

Abnormal epiphyseal growth plate development of the proximal tibia in hemophilia patients leads to notable morphological changes in the mature knee joint. This study aimed to compare the morphological characteristics of tibial component placement cut surface in patients with hemophilic arthritis (HA) and osteoarthritis (OA) and to determine the tibial component rotational alignment axis’ best position for HA patients.

**Methods:**

Preoperative computed tomography scans of 40 OA and 40 HA patients who underwent total knee arthroplasty were evaluated using a three-dimensional (3D) software. The tibial component’s placement morphological parameters were measured. The tibial component’s rotational mismatch angles were evaluated, and the most appropriate 0°AP axis position for HA patients was investigated.

**Results:**

In the two groups, the morphology was significantly different in some of the parameters (*p* < 0.05). The tibial component rotational mismatch angles were significantly different between both groups (*p* < 0.05). The medial 9.26° of the medial 1/3 of the patellar tendon was the point through which 0°AP axis passed for the HA patients. Similarly, the medial 13.02° of the medial 1/3 of the tibial tubercle was also the point through which the 0°AP axis passed.

**Conclusions:**

The ratio of the anteroposterior length to the geometric transverse length of the placement section of the tibial component in HA patients was smaller than that in OA patients. The medial 9.26° of the medial 1/3 of the patellar tendon or the medial 13.02° of the medial 1/3 of the tibial tubercle seem to be an ideal reference position of the rotational alignment axis of the tibial component for HA patients.

## Introduction

**Fig. 1:**
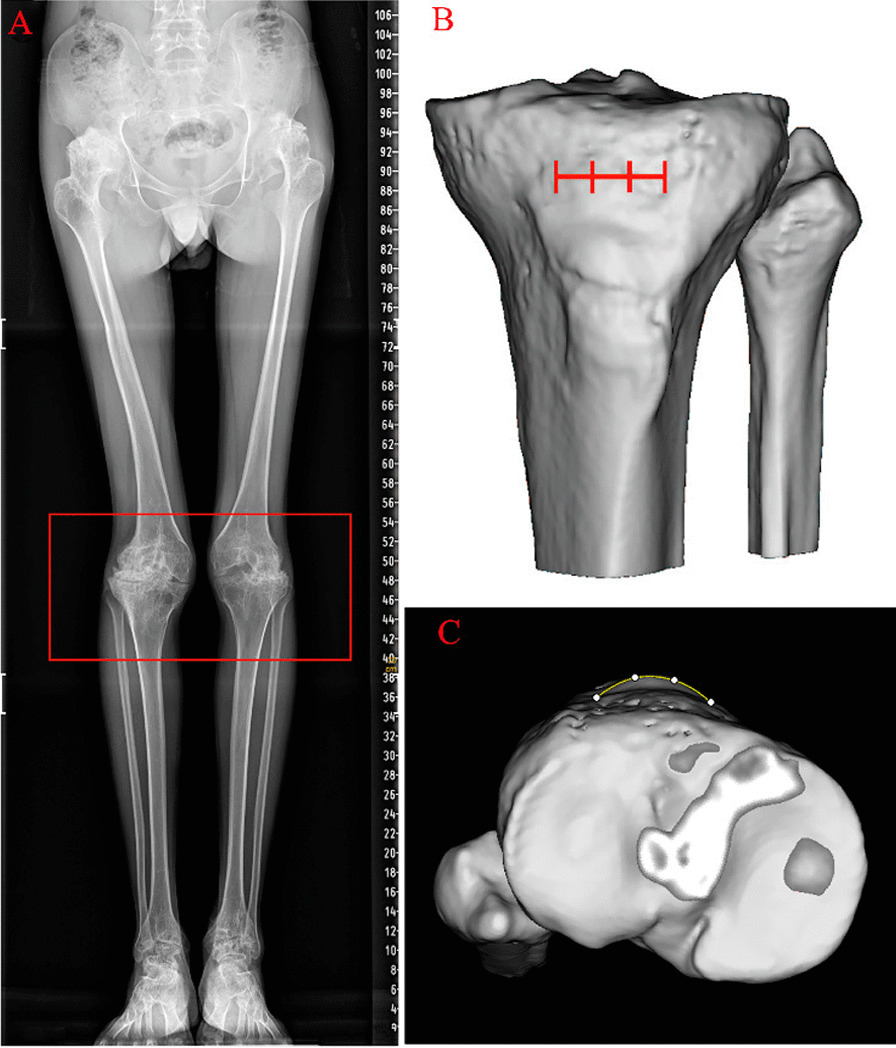
**A** The degree of injury of the hip, knee, and ankle joints in hemophiliacs, especially the knee joints. **B**, **C** The tibial tubercle is equally divided into three equal parts on the axial and top views

Hemophilia A and B are X-linked recessive disorders which result in lack of clotting factors VIII and IX, respectively [1]. They are classified as rare diseases; the worldwide prevalence of hemophilia A and B in men is approximately 1/5000 and 1/30,000, respectively [2]. Hemophilic arthropathy (HA) is the most common joint complication in patients with hemophilia [3]. Symptoms begin during early childhood and last throughout the person’s lifetime. Due to the lack of coagulation factors in the body, the joint spaces are prone to recurrent spontaneous bleeding [4]. With continuous deposition of iron in the blood and ongoing damage to the articular synovium and cartilage, chronic proliferative synovitis and destruction of articular cartilage ensue [5, 6]. This is detrimental for the development of immature joints, particularly at the epiphyseal growth plate [7]. With increasing age, morphological and anatomical changes in adult joint bone result from continuous effect of synovitis on the epiphyseal growth plate and ongoing damage by iron to the articular cartilage [8]. This process is difficult to reverse, even with early coagulation factor replacement therapy [9]. The knee, hip, ankle, and elbow joints of these patients are the most commonly injured regions, among which the knee joint injury is the most prevalent [10] (Fig. 1A).

Due to asymmetric growth of the epiphysis, abnormal proliferation of synovial tissue, and degenerative changes in articular cartilage, the knee joint is characterized by a developmental deformity of the tibial plateau, a decrease in the aspect ratio of the platform, flat intercondylar eminence, giant osteophyte formation of the tibial plateau in the early stage, variation in tibial tubercle position, and square patella formation [11, 12]. Therefore, it is difficult for joint surgeons to accurately determine the anatomical landmarks of the rotational alignment of the tibial component on the proximal tibia; it is difficult to perform the precise component placement and rotational alignment between the prostheses during total knee arthroplasty (TKA). Previous studies on the position of the tibial component’s rotational alignment have mostly focused on osteoarthritis (OA) of the knee [13–16]. However, the best position of the tibial component’s rotational alignment axis for HA patients and whether the tibial component can be placed according to the rotational alignment standard of the tibial prosthesis in OA patients have not been studied. Therefore, we investigated the morphological characteristics of the tibial component placement section and the difference in position of the rotational alignment axis in the two groups of patients.

This study aimed to compare the morphological differences in the proximal tibia section between male OA and HA patients. Additionally, we sought to ascertain the difference in the position of the rotational alignment axis of the tibial component using the same method to determine the best position of the rotational alignment axis of the tibial component in HA patients.

## Materials and methods

### Patients

**Fig. 2:**
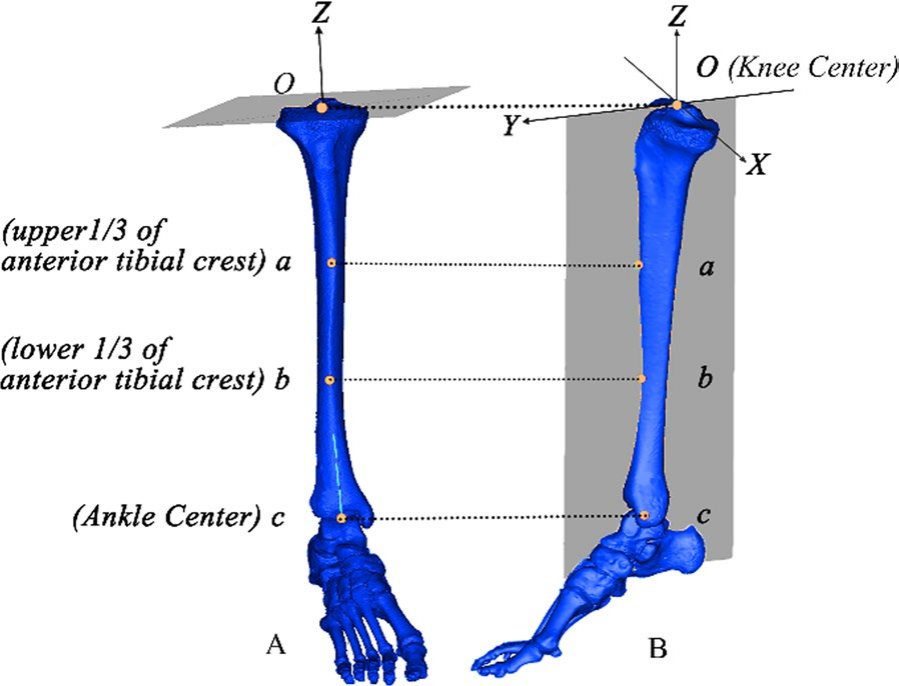
The coordinate system on the tibial model (**A**, **B**). X-,Y-,Z-axes are perpendicular to each other

We retrospectively screened 40 knees in 40 male patients with HA and end-stage hemophilic knee arthritis who underwent detailed registration and clinical treatment at our hospital between January 2017 and December 2020. The patients were assessed as follows: inclusion criteria—radiologically confirmed end-stage degenerative knee arthritis, severe dysfunction of knee joint movement, persistent knee joint pain, ineffective conservative treatment, and obvious indications for total knee arthroplasty; exclusion criteria—severe knee varus or valgus deformity (deformity angle > 20°) [17], patellar dislocation, previous knee surgery or joint platform fracture, knee joint congenital dysplasia, knee joint osseous fusion, and presence of the knee tumor. The 40 HA patients we screened were aged 20–55 years, had radiographic Arnold-Hilgartner grade IV, and lacked factor VIII inhibitor (Fig. 2).

In order to make a comparative study, we selected OA patients as controls among the 305 OA patients who underwent TKA in our hospital during the same period. According to the matching conditions of male and end-stage degenerative knee arthritis with the radiographical grade [Kellgren-Lawrence (KL)]: grade 3–4, 105 OA patients who met the inclusion criteria and exclusion criteria were selected, and then matched 1:1 according to the number of HA patients included. Finally, 40 OA (42 knees) were selected as the control group. There was no significant difference in demographic data (Height, BMI, Radiological grade, Side) between the two groups of patients (*P* > 0.05), and a comparative study was conducted. The participants’ demographic data are presented in Table 1.

**Table 1:** Demographic data of the HA and OA groups

Patient demographics	HA group (40 knees)	OA group (42 knees)	*P*
Age (years)ª	32.48 ± 10.25	62.31 ± 11.34	0.001
Height^a^ (cm)	172.09 ± 2.05	170.87 ± 6.66	0.271
BMIª	23.79 ± 2.82	24.03 ± 3.80	0.747
KL grade			0.241
	KL grade 4 (40 knees)	KL grade 3(3 knees)	
		KL grade 4 (39 knees)	
Side (Lt/Rt)			0.991
	Lt: 21 knees	Lt: 22 knees	
	Rt: 19 knees	Rt: 20 knees	
*Deformity angle*^*a*^ (°)			
Varus	3.36 ± 2.17 (17 knees)	11.23 ± 5.90 (35 knees)	0.001
Valgus	7.50 ± 6.42 (23 knees)	7.14 ± 6.09 (7 knees)	0.897

*BMI*: Body Mass Index, *HA*: Hemophiliac Arthritis, *KL*: Kellgren-Lawrence, *Lt*: left, *OA*: Osteoarthritis, *Rt*: right

^a^The value is given as the mean ± SD

### Equipment and methods of measurement

**Fig. 3:**
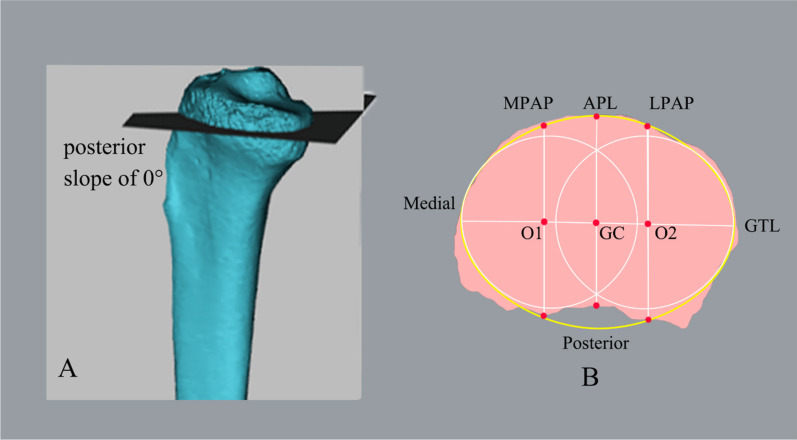
**A** 3D model of the proximal tibial cut 8 mm below the lateral platform center with a posterior slope of 0°. **B** Morphology parameters of the osteotomy surface. MPAP and LPAP; the anteroposterior diameter of the medial and lateral platform of the osteotomy plane. APL; anteroposterior length of the osteotomy plane. O1 and O2; the centers of medial and lateral platform of the osteotomy plane, respectively. GC; the center of the oval shape that best matches the edge outline of the cutting surface. GTL; geometric transverse length of the osteotomy plane. APL; anteroposterior length of the osteotomy plane

The computed tomography (CT) of the knee joint was scanned using the spiral scanning mode of the LightSpeed 64 row volume CT (VCT) machine (General Electric LightSpeed VCT, GE Healthcare, Milwaukee, WI, USA; image array 512 × 512, fastest time resolution 43 ms, contrast resolution 5 mm@ 0.3%, spatial resolution 15.42lp/cm). During imaging, the patient remained in the supine position with the knee joint straightened as much as possible. The scanning range was the ipsilateral lower extremity, starting from 10 cm upper the tibial plateau. The slice thickness of each row of detectors is 0.625 mm. A three-dimensional (3D) image of the knee joint was reconstructed using a 3D reconstruction computer program (Post-processing 3D workstation, IntelliSpace Portal v6.0.5.02900; Philips Company, Germany). Finally, the original data scanned by the CT were copied to a Digital Imaging and Communications in Medicine optical disk in DICOM format and entered into Mimics Medical 21.0 (Materialise, Leuven, Belgium), to reconstruct the 3D bone model of the lower extremity. The 3D objects were exported to 3-matics in order to simulate the surgical procedures and the proximal tibial cut at a 0° posterior slope angle [13] (Fig. 3A).

We defined the medial and lateral platform center of the knee joint, the knee center and the ankle center according to the research by Ma et al. [18]. The knee center was defined as the origin of the coordinate and the tibial mechanical axis was defined as the Z-axis which connects the knee and ankle centers. The Y-axis was defined as the line connecting the knee center to the point closest to the two points (upper and lower 1/3 of anterior tibial crest) on the normal plane of tibial mechanical axis, and the X-axis was defined as the axis perpendicular to both the Y- and Z-axes. Finally, the coordinate system was established using the findings by Cobb et al. [19] , Dai et al. [20] and Ma et al. [13] as reference (Fig. 2).

**Fig. 4:**
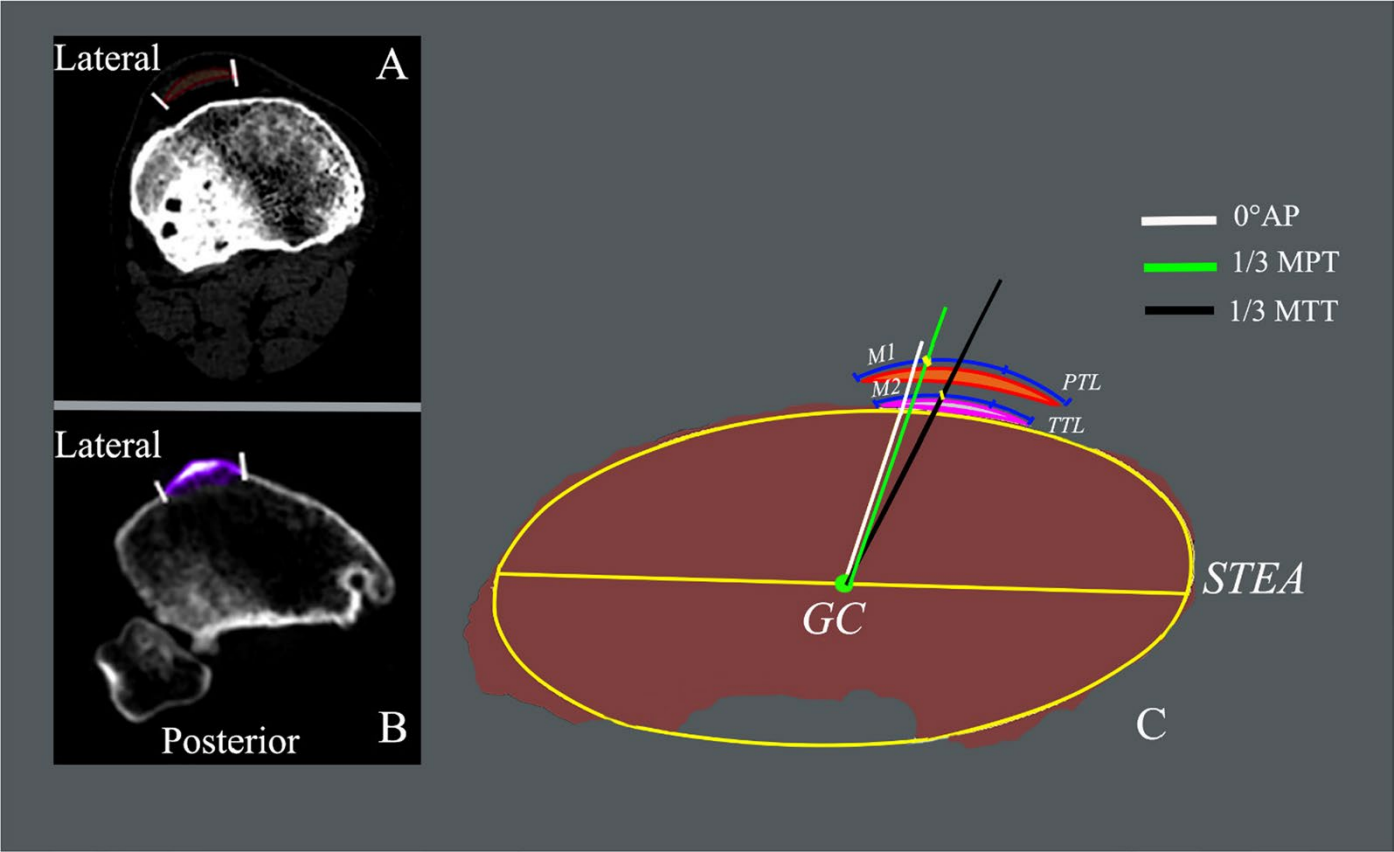
**A**, **B** Patellar tendon and tibial tubercle on CT axial cut. **C** The angle between the 1/3MPT, 1/3MTT axis, and the 0°AP axis was defined as the rotational mismatch angle. PTL; patellar tendon length. TTL; tibial tubercle length. M1: width of patellar tendon on the medial side of 0°AP axis. M1/PTL; the medial patellar tendon width percentage. M2; width of the tibial tubercle on the medial side of 0°AP axis. M2/TTL; the medial tibial tubercle width percentage. GC; geometric center. 1/3MPT; medial one-third of the patellar tendon. 1/3MTT; medial one-third of the tibial tubercle

The surgical transepicondylar axis (STEA) is the functional flexion and extension axis of the knee joint [21]. Therefore, for patients who undergo TKA, the rotational alignment axis of the tibial prosthesis should be as consistent as possible with the STEA projection line or its vertical line (0° anterior–posterior (0° AP) axis) on the tibial cut surface as far as possible to achieve ideal rotational alignment between the implants. However, because the STEA projection line cannot be accurately identified, other landmarks on the section were used to guide the rotational alignment of the tibial prosthesis during TKA. The medial one-third of the patellar tendon (1/3MPT) [18] is a commonly used landmark, and its connecting line to the geometric center (GC) of the tibial section can be used as the rotation alignment axis of the tibial prosthesis (Fig. 4C). Therefore, we verified the accuracy of this axis in patients with HA. We also investigated the morphological parameters of the proximal tibia section and related parameters of the prosthesis rotational alignment in male OA and HA patients. In a previous study [14], the 1/3MPT was used instead of the medial one-third of the tibial tubercle (1/3MTT); therefore, the connecting line between the 1/3MTT and GC was also used to evaluate the rotation alignment of the tibial prosthesis between the two groups (Fig. 4C).

Measurement plane was selected on the proximal tibia at the level of 8 mm below lateral plane center to simulate the 10 mm osteotomy thickness during the TKA [22], which was determined considering approximately 2 mm lateral cartilage referred from the previous papers [23]. The GC of the plane was defined as the oval center, which was the most suitable for the outline of the cutting surface [15, 18]. On the selected cutting surface, the GC was set as the rear endpoint of the rotational alignment axis, and STEA was selected and passed through the GC where the 0°AP axis passed through the GC and was perpendicular to the STEA. The GC was connected to the 1/3MPT (the connection line was set to the1/3MPT axis) and 1/3MTT (the connection line was set to the 1/3MTT axis) as the two original AP axes (Fig. 4). The angle between the 0°AP axis and 1/3MPT or 1/3MTT axis was the rotation mismatch angle of the prosthesis. Ideally, 1/3MPT and 1/3MTT axis should be consistent with the 0°AP axis.

### Observation indicators

The angle between 1/3MPT axis and 0°AP axis, patellar tendon length (PTL), patellar tendon medial width of the 0°AP axis crossing the patellar tendon (M1), medial percentage length (M1/PTL × 100%), angle between the 1/3MTT axis and 0°AP axis, tibial tubercle length (TTL), tibial tubercle medial width of the 0°AP axis crossing the tibial tubercle (M2), medial length percentage (M2/TTL × 100%), anteroposterior diameter of the medial and lateral platform of the osteotomy plane (MPAP; LPAP), geometric transverse length (GTL), anteroposterior length (APL) of the osteotomy surface, and the ratio of APL and GTL (APL/GTL) were the observation indicators (Fig. 3). The study included 80 patients, and all participants were fully aware of the CT inspection and they signed an informed consent and risk notification forms. The institutional review committee approved the study, protocol number: YX 2021–071 (F1), approval date: 6 July, 2021.

### Statistical analysis

All data were statistically analyzed using SPSS 25.0 (IBM, Inc., Armonk, NY, USA). The data of both groups are presented as the mean ± standard deviation (SD) for continuous variables, which were compared by an independent sample *t* test; and as numbers for categorical variables, which was compared by *χ*^2^ test and Fisher's exact test, *P* < 0.05 was considered statistically significant.

## Results

For morphological variables of the selected section, there were significant differences in MPAP, LPAP, APL, PTL, M1, and M2 between the two groups (*p* < 0.05), but there was no significant difference in GTL and TTL (*p* > 0.05) (Table 2). The results showed that the medial platform of the osteotomy surface was larger than the lateral platform in the HA and OA groups, and this feature was more prominent in the HA group. Additionally, the values of the APL, PTL GTL, M1, and M2 in the OA group were significantly higher than those in the HA group, but the TTL of the OA group was significantly lower than that of the HA group.

**Table 2:** Morphological variables of the selected section (mm)

Morphological variables	HA group (*n* = 40) Mean ± SD (range)	OA group (*n* = 42) Mean ± SD (range)	*t*	*P*
MPAP	45.26 ± 3.96 (36.94, 55.63)	49.78 ± 3.06 (42.74, 56.47)	− 5.808	0.000
LPAP	41.39 ± 5.426 (31.29, 52.69)	47.62 ± 4.49 (40.15, 56.69)	− 5.689	0.000
GTL	74.21 ± 5.22 (61.01, 86.54)	74.79 ± 5.90 (55.31, 82.19)	− 0.474	0.636
APL	46.60 ± 5.00 (33.36, 54.39)	49.63 ± 3.41 (41.40,56.21)	− 3.200	0.002
PTL	24.47 ± 3.58 (18.86, 36.00)	27.52 ± 2.68 (22.60, 32.78)	− 4.391	0.000
TTL	24.40 ± 3.35 (15.87, 31.82)	23.22 ± 3.14 (16.13, 30.83)	1.652	0.102
M1	3.66 ± 3.98 (− 2.51^b^, 18.32)	7.59 ± 4.35 (− 3.07^b^, 14.51)	− 4.269	0.000
M2	3.07 ± 4.01 (− 4.56^b^, 15.20)	4.90 ± 3.67 (− 0.81^b^, 16.90)	− 2.154	0.034

*HA*: Hemophiliac Arthritis, *OA*: Osteoarthritis, *SD*: Standard Deviation, *MPAP*: the anteroposterior diameter of the medial platform of the osteotomy plane, *LPAP*: the anteroposterior diameter of the lateral platform of the osteotomy plane, *GTL*: geometric transverse length of the osteotomy plane, *APL*: anteroposterior length of the osteotomy plane, *PTL*: patellar tendon length, *TTL*: tibial tubercle length, *M1*: width of patellar tendon on the medial side of 0°AP axis, *M2*: width of the tibial tubercle on the medial side of 0°AP axis

^b^Negative values indicate the medial areas of the medial edge of the patellar tendon or tibial tubercle

Regarding the rotating mismatch angle of the original AP axis, the 1/3MPT and 1/3MTT axes in both groups showed obvious external rotation angles compared with the 0°AP axis. There was a significant difference between both groups (*p* < 0.05). The angle of external rotation of the 1/3MPT and 1/3MTT axis in the HA group was more obvious than that in the OA group, and the degree of external rotation of the 1/3MTT axis in the HA group was more obvious than that of the 1/3MPT axis (the 1/3MTT axis: 13.02°, the 1/3MPT axis: 9.26°). Similarly, the angle of external rotation of the 1/3MTT axis was more distinct than that of the 1/3MPT axis (the 1/3MTT axis: 6.42°, the 1/3MPT axis: 3.15°) in the OA group (Table 3).

**Table 3:** The angle between the original AP axis (AP_1_, AP_2_) and 0°AP axis

Angle	HA group(*n* = 40) Mean ± SD(range)	OA group(*n* = 42) Mean ± SD (range)	*t*	*P*-value
The angle between 1/3MPT axis and 0°AP axis	+ 9.26° ± 6.31° (− 1.71°, + 27.05°)	+ 3.15° ± 5.62° (− 13.68°, + 16.34°)	4.626	0.000
The angle between 1/3MTT axis and 0°AP axis	+ 13.02° ± 7.18° (− 7.00°, + 25.19°)	+ 6.42° ± 5.44° (− 8.42°, + 16.85°)	4.702	0.001

The original 1/3MPT axis and 1/3MTT axis external rotation was set to + and internal rotation to—relative to the 0°AP axis

*HA*: Hemophiliac Arthritis, *OA*: Osteoarthritis, *SD*: Standard Deviation, *1/3MPT*: the medial one-third of the patellar tendon, *1/3MTT*: the medial one-third of the tibial tubercle

There were significant differences in APL/GTL, M1/PTL, and M2/TTL between both groups (*p* < 0.05), and the ratios of the OA group were higher than those of the HA group (Table 4). These results indicate that the 0°AP axis of the OA group is more lateral to the medial edge of the patellar tendon than the 0°AP axis of the HA group. The position of the 0°AP axis of the OA group was in the medial 1/4 to 1/3 range of the patellar tendon and approached the medial 1/4, while the 0°AP axis of the HA group was in the medial 1/7 to 1/6 range of the patellar tendon and approached the medial 1/7. Similarly, the M2 in the HA and OA groups accounted for 12.23% and 20.82% of the TTL, respectively. These are located in the range of the medial 1/9 to 1/8 and medial 1/5 to 1/4 of the tibial tubercle, respectively, and approached the medial 1/ 8 and 1/5 points of the tibial tubercle, respectively.

**Table 4:** Ratios of related parameters of the osteotomy surface

Proportions	HA group (*n* = 40) Mean ± SD (range)	OA group (*n* = 42) Mean ± SD (range)	*t*	*P*
APL/GTL	0.6265 ± 0.0497 (0.5019, 0.7105)	0.6677 ± 0.0695 (0.5574, 0.8972)	− 3.072	0.003
M1/PTL	0.146 ± 0.1607 (− 0.1061^c^, 0.7743)	0.2686 ± 0.1479 (− 0.1288^c^, 0.5106)	− 3.586	0.001
M2/TTL	0.1223 ± 0.1612 (− 0.2037^c^, 0.5815)	0.2082 ± 0.1574 (− 0.0373^c^, 0.6812)	− 2.441	0.017

*HA*: Hemophiliac Arthritis, *OA*: Osteoarthritis, *SD*: Standard Deviation, *APL/GTL*: the ratio of the anteroposterior length to the geometric transverse length of the osteotomy surface, *M1/PTL*: the medial patellar tendon width percentage, *M2*: width of the tibial tubercle on the medial side of 0°AP axis, *M2/TTL*: the medial tibial tubercle width percentage

^c^The value is assigned as the 0°AP axis on the internal of the medial edge of the patellar tendon or tibial tubercle

## Discussion

The most important finding of this study is that the morphology of the proximal tibial section of HA patients is significantly different from that of the OA patients, and that the reference landmark’s position of the rotational alignment of the tibial prosthesis in OA patients is not suitable for HA patients. Morphologically, the proximal tibial section of the HA patients was smaller compared with that of the OA patients, as was the ratio of the anteroposterior length to the mediolateral width of the placement section of the tibia (Tables 2 and 4). Therefore, it is suggested that due to the early onset of disease in patients with HA, the knee joint is damaged by blood in the articular cavity over a prolonged period, affecting the development of the epiphyseal growth plate and finally leads to local morphological and anatomical changes of the proximal tibial section in adulthood [7, 8, 24]. For patients with HA, the 1/3MPT and 1/3MTT axes, respectively, would cause excessive external rotation of the prosthesis (Table 3). However, the external rotational angle of the prosthesis is notably smaller when the 1/3MPT axis is used to determine the rotational alignment relative to the 1/3MTT axis. This reveals the considerable variability of the tibial tubercle’s position in the rotational alignment of the tibial component [25]. Our results indicated that when the 1/3MPT axis was used to determine the rotational alignment of the prosthesis, the external rotation in the OA group was only 3.15°, which is consistent with the results of Ma et al. [18] in OA patients. Therefore, in the background of this study method, it seems reasonable to use 1/3MPT as the reference landmark for the tibial component’s rotational alignment.

It is established that the prosthesis’ direction of rotational alignment is determined by the anatomical landmarks on the osteotomy plane. Therefore, for HA patients, changes in anatomy caused by morphological variations may make it difficult for surgeons to accurately determine the position of rotational alignment axes, thereby making it challenging to determine the rotational alignment of the tibial prosthesis [26–28].

Theoretically, the optimum rotational alignment between the prostheses is such that the STEA or 0°AP axis and the rotation alignment axis of the tibial prosthesis coincide with each other. The perfect overlap of the AP axis of the tibial prosthesis and 0°AP axis of the tibial section is important in achieving accurate alignment of the prosthesis. Therefore, it is crucial to select an appropriate reference landmark to determine the prosthesis’ rotational alignment axis. The results of this study indicate that the 1/3MPT is relatively more accurate than the 1/3MTT because the rotational alignment axis determined by 1/3MTT produced a greater rotational mismatch angle of the prosthesis than that obtained when 1/3MPT is used in both groups. Therefore, it is more clinically instructive to choose the 1/3MPT as the landmark for rotational alignment [18]. However, the axis determined by 1/3MPT is not suitable for the HA patients because it may produce an average tibial prosthesis external rotation of 9.26° and maximum external rotation of 27.05°, which are significantly beyond the acceptable range of rotation mismatch of 2°–5° [25]. Our study shows that the ideal position of the rotation alignment axis for the HA patients should be located at the medial 9.26° of the 1/3MPT (lateral 3.67 mm from the medial edge), or in the range of the medial 1/7 to 1/6 of the patellar tendon and approaching the 1/7 point (M1/PTL = 14.61%).

Currently, although several reference landmarks for the rotational alignment of tibial prostheses have been proposed, there are varying degrees of deviation in clinical use [29]. The 1/3 MTT is considered to be a classic landmark of rotational alignment, but it could lead to a greater degree of rotation mismatch of the tibial prosthesis [14] and the medial border of the tibial tubercle [25]. We believe this is related to the fact that the position of the tibial tubercle is prone to variation and its medial and lateral edges cannot be accurately identified [14, 25, 30, 31]. However, most surgeons still use it as a reference to determine the direction of rotational alignment of the prosthesis because it provides higher survival rate of the prosthesis and relatively lower postpatellar pressure compared to other reference landmarks after TKA [32–34].

The rotational alignment of the prosthesis is only one of the knee joint’s conditions for functional recovery after TKA. Therefore, providing the maximum coverage area and ensuring the prosthesis’ rotational alignment while not exceeding the outline of the cutting surface is the primary consideration during placement of the tibial prosthesis [16, 18]. We believe that the greater coverage area of the prosthesis and more adequate contact between the prosthesis and cortical bone, the lower the contact stress of the bone–implant interface. Therefore, the prosthesis can provide optimum stability and the transfer of lower limb stress load, to avoid subsidence and loosening of the prosthesis [35, 36]. This condition is particularly important for HA patients because they have long-term osteoporosis, bone defects, and subchondral bone cysts [6]. Additionally, the low coverage rate of the prosthesis will result in abnormal conduction of the lower limb stress, resulting in periprosthetic fracture and even loosening [36, 37]. Based on these factors, we recommend the use of anatomical prostheses with larger coverage because they can better match the shape of the tibial section and effectively prevent the rotation mismatch between the prostheses [18, 38].

Our study has several limitations. First, the patients’ ages were not perfectly matched between the groups. However, given that the HA patients presented with similar knee joint lesions and symptoms when at a younger age as senior OA patients or even exhibited more severe knee joint degeneration than the OA patients, and they were relatively younger to undergo primary TKA, the individual factors of this disease were not taken into consideration. Second, this study only comparatively studied the knee joints of OA and HA patients and did not introduce the knee joints of healthy volunteers as the control group for comparison. Third, the results of our study may not apply to the severe varus or valgus deformed knee joints because they were excluded from the study in the first step. Fourth, our study had an insufficient sample size. Finally, our results are limited to theoretical research and lacks the support of clinical validation data. This will be addressed in the next step of this research. Nevertheless, the results of the study should be able to provide guidance for surgeons for dealing with knee joints of HA patients with significant morphological changes.

## Conclusions

The morphological changes in the placement section of the tibial prosthesis led to the change in position of the rotational alignment axis of the tibial prosthesis. The medial 9.26° of 1/3MPT (lateral 3.67 mm from the medial edge) or the medial 13.02° of 1/3MTT (lateral 3.07 mm from medial edge) is an ideal reference position for the rotation alignment axis of the tibial prosthesis in HA patients, which are in the medial 1/7 to 1/6 range of the patellar tendon while approaching the 1/7 point or the medial 1/9 to 1/8 range of the tibial tubercle while approaching 1/8 point.

## References

[1] Pasta G, Vanelli R, Jannelli E (2018). Primary total knee replacement in hemophiliacs: experience of a single institution over fourteen years of surgical procedures. J Biol Regul Homeost Agents.

[2] Berntorp E, Shapiro AD (2012). Modern haemophilia care. Lancet.

[3] Pasta G, Jannelli E, Ivone A (2020). The role of six biomarkers in diagnosis of hemophilic arthropathy: review of the literature. J Biol Regul Homeost Agents.

[4] Chozie NA, Primacakti F, Gatot D (2019). Comparison of the efficacy and safety of 12-month low-dose factor VIII tertiary prophylaxis vs on-demand treatment in severe haemophilia A children. Haemophilia.

[5] Zhu H, Meng Y, Tong P (2021). Pathological mechanism of joint destruction in haemophilic arthropathy. Mol Biol Rep.

[6] Pasta G, Annunziata S, Polizzi A (2020). The progression of hemophilic arthropathy: the role of biomarkers. Int J Mol Sci.

[7] Rodriguez-Merchan EC (1996). Effects of hemophilia on articulations of children and adults. Clin Orthop Relat Res.

[8] Hamel J, Pohlmann H, Schramm W (1988). Radiological evaluation of chronic hemophilic arthropathy by the Pettersson score: problems in correlation in adult patients. Skeletal Radiol.

[9] Bhat V, Olmer M, Joshi S (2015). Vascular remodeling underlies rebleeding in hemophilic arthropathy. Am J Hematol.

[10] Liddle AD, Rodriguez-Merchan EC (2017). Evidence-based management of the knee in hemophilia. JBJS Rev.

[11] Arnold WD, Hilgartner MW (1977). Hemophilic arthropathy. Current concepts of pathogenesis and management. J Bone Joint Surg Am.

[12] Nacca CR, Harris AP, Tuttle JR (2017). Hemophilic arthropathy. Orthopedics.

[13] Ma Y, Mizu-Uchi H, Ushio T (2019). Bony landmarks with tibial cutting surface are useful to avoid rotational mismatch in total knee arthroplasty. Knee Surg Sports Traumatol Arthrosc.

[14] Uehara K, Kadoya Y, Kobayashi A (2002). Bone anatomy and rotational alignment in total knee arthroplasty. Clin Orthop Relat Res.

[15] Kawahara S, Okazaki K, Matsuda S (2014). Medial sixth of the patellar tendon at the tibial attachment is useful for the anterior reference in rotational alignment of the tibial component. Knee Surg Sports Traumatol Arthrosc.

[16] Hirakawa M, Miyazaki M, Ikeda S (2017). Evaluation of the rotational alignment of the tibial component in total knee arthroplasty: position prioritizing maximum coverage. Eur J Orthop Surg Traumatol.

[17] De Muylder J, Victor J, Cornu O (2015). Total knee arthroplasty in patients with substantial deformities using primary knee components. Knee Surg Sports Traumatol Arthrosc.

[18] Ma Y, Mizu-Uchi H, Okazaki K (2018). Effects of tibial baseplate shape on rotational alignment in total knee arthroplasty: three-dimensional surgical simulation using osteoarthritis knees. Arch Orthop Trauma Surg.

[19] Cobb JP, Dixon H, Dandachli W (2008). The anatomical tibial axis: reliable rotational orientation in knee replacement. J Bone Joint Surg Br.

[20] Dai Y, Scuderi GR, Bischoff JE (2014). Anatomic tibial component design can increase tibial coverage and rotational alignment accuracy: a comparison of six contemporary designs. Knee Surg Sports Traumatol Arthrosc.

[21] Mantas JP, Bloebaum RD, Skedros JG (1992). Implications of reference axes used for rotational alignment of the femoral component in primary and revision knee arthroplasty. J Arthroplasty.

[22] Li G, Park SE, Defrate LE (2005). The cartilage thickness distribution in the tibiofemoral joint and its correlation with cartilage-to-cartilage contact. Clin Biomech.

[23] Matsuda S, Miura H, Nagamine R (2004). Anatomical analysis of the femoral condyle in normal and osteoarthritic knees. J Orthop Res.

[24] Liu S, Zhou RF, Jin ZB (2020). Age-related severity and distribution of haemophilic arthropathy of the knee, ankle and elbow among Chinese patients with haemophilia. Haemophilia.

[25] Howell SM, Chen J, Hull ML (2013). Variability of the location of the tibial tubercle affects the rotational alignment of the tibial component in kinematically aligned total knee arthroplasty. Knee Surg Sports Traumatol Arthrosc.

[26] Van Gennip S, Schimmel JJ, Van Hellemondt GG (2014). Medial patellofemoral ligament reconstruction for patellar maltracking following total knee arthroplasty is effective. Knee Surg Sports Traumatol Arthrosc.

[27] Kim JI, Jang J, Lee KW (2017). Anterior tibial curved cortex is a reliable landmark for tibial rotational alignment in total knee arthroplasty. BMC Musculoskelet Disord.

[28] Mannan A, Smith TO (2016). Favourable rotational alignment outcomes in PSI knee arthroplasty: a level 1 systematic review and meta-analysis. Knee.

[29] Akagi M, Mori S, Nishimura S (2005). Variability of extraarticular tibial rotation references for total knee arthroplasty. Clin Orthop Relat Res.

[30] Tao K, Cai M, Li SH (2010). The anteroposterior axis of the tibia in total knee arthroplasty for chinese knees. Orthopedics.

[31] Hovinga KR, Lerner AL (2009). Anatomic variations between Japanese and Caucasian populations in the healthy young adult knee joint. J Orthop Res.

[32] Kim YH, Park JW, Kim JS (2014). The relationship between the survival of total knee arthroplasty and postoperative coronal, sagittal and rotational alignment of knee prosthesis. Int Orthop.

[33] Lutzner J, Krummenauer F, Gunther KP (2010). Rotational alignment of the tibial component in total knee arthroplasty is better at the medial third of tibial tuberosity than at the medial border. BMC Musculoskelet Disord.

[34] Steinbruck A, Schroder C, Woiczinski M (2016). Influence of tibial rotation in total knee arthroplasty on knee kinematics and retropatellar pressure: an in vitro study. Knee Surg Sports Traumatol Arthrosc.

[35] Huang C-H, Cheng C-K, Liau J-J, Lee Y-M (2000). Morphometrical comparison between the resected surfaces in osteoarthritic knees and porous-coated anatomic knee prosthesis. J Musculoskelet Res.

[36] Bourne RB, Finlay JB (1986). The influence of tibial component intramedullary stems and implant-cortex contact on the strain distribution of the proximal tibia following total knee arthroplasty. An in vitro study. Clin Orthop Relat Res.

[37] Mortazavi SJ, Bagheri N, Farhoud A (2020). Total knee arthroplasty in patients with hemophilia: What do we know?. Arch Bone Jt Surg.

[38] Stulberg SD, Goyal N (2015). Which tibial tray design achieves maximum coverage and ideal rotation: Anatomic, symmetric, or asymmetric? An MRI-based study. J Arthroplast.

